# Level of clinical evidence presented at the International Society for Hip Arthroscopy Annual Scientific Meeting over 5 years (2010–2014)

**DOI:** 10.1093/jhps/hnv059

**Published:** 2015-08-14

**Authors:** Jeffrey Kay, Darren de SA, Scott Shallow, Nicole Simunovic, Marc R. Safran, Marc J. Philippon, Olufemi R. Ayeni

**Affiliations:** 1. Michael G. DeGroote School of Medicine,; 2. Division of Orthopaedic Surgery, Department of Surgery,; 3. Department of Clinical Epidemiology and Biostatistics, McMaster University, Hamilton, Ontario, Canada,; 4. Division of Orthopedic Surgery, Stanford University, Stanford, CA, USA and; 5. Steadman Philippon Research Institute, Vail, CO, USA

## Abstract

The International Society for Hip Arthroscopy (ISHA) Annual Scientific Meeting is at the forefront of informing today’s orthopaedic surgeons and society of the rapid advances in the exponentially growing field of hip arthroscopy. The purpose of this study was to evaluate and observe any trends in the level of clinical evidence in the papers and posters presented at the ISHA Annual Scientific Meeting from 2010 to 2014. The online abstracts of the paper and poster presentations presented at the ISHA Annual Scientific Meetings were independently evaluated by two reviewers (582 total resulting presentations). Two reviewers screened these results for clinical studies and graded the quality of evidence from level I (i.e. randomized trials) to IV (i.e. case series) based on the American Academy of Orthopaedic Surgeons classification system. Four hundred and twenty-eight presentations met the inclusion criteria and were evaluated. Overall, 10.1% of the presentations were level I, 12.8% were level II, 30.1% were level III and 47.0% were level IV evidence. Over time, from 2010 to 2014, we observed an increase in the percentage of level II paper presentations, an increase in the proportion of level III poster presentations, and a decrease in the proportion of both level IV paper and poster presentations. Significant non-random improvement in the level of evidence presented was noted for the poster presentations (*P* = 0.012) but not for the paper presentations (*P* = 0.61) over the study period. Statistical trends demonstrate ISHA’s increased awareness and commitment to presenting higher quality evidence as the availability of this evidence increases.

## INTRODUCTION

The use of hip arthroscopy as a means of addressing central and peripheral compartment pathology has increased substantially in the last few years—both in the number of procedures being performed and the number of individuals performing such procedures [1]. One study has reported an 18-fold increase in the number of arthroscopic hip procedures performed by young orthopaedic surgeons between 1999 and 2009 [2]. The increase in the number of surgeons specializing in this field has led to the creation of the International Society for Hip Arthroscopy (ISHA) in 2008. In 2009 the first ISHA Annual Scientific Meeting took place in New York City and since its inaugural event, has continued annually on a global scale. Due to the relative novelty of hip arthroscopy, these meetings are an important source of information for surgeons in this specialty, with the potential to influence clinical decisions and health policy.

In order to provide proper, well-founded information to physicians that attend this scientific meeting, it is important to select study designs higher on the hierarchy of evidence. Sackett *et al*. [3, 4] introduced the critical concepts of Evidence-Based Medicine (EBM) and have emphasized understanding and improving the quality of research that is presented. The American Academy of Orthopaedic Surgeons (AAOS) has standardized this approach for research in orthopaedic surgery by creating an evaluation system adopted from the system used by *The Journal of Bone and Joint Surgery* [5]. This system assigns a particular level of evidence (from I to IV) based on study design with randomized controlled trials (RCTs) deemed level I evidence and case series or reports deemed level IV evidence. The idea of this classification system is that a more rigorous study design would inherently have an outcome that is more reliable in terms of its clinical applications.

There are various determinants of the quality of research presented at a scientific meeting. Because the field of arthroscopic hip surgery is relatively novel, producing high-quality evidence with rigorous methodology could be difficult, particularly when studying new procedures. The purpose of this study was to evaluate the level of evidence of both the poster and the paper presentations presented at the ISHA Annual Scientific Meeting for the years 2010 through to 2014. Second, we aimed to evaluate if any changes in trends existed with regards to level of evidence presented over time.

## MATERIALS AND METHODS

### Eligibility and analysis of presentations

Eligible presentations included clinical article and poster presentations presented at the 2010, 2011, 2012, 2013 and 2014 ISHA Annual Scientific Meeting. Clinical research includes trials and observational studies where there is a direct interaction between an investigator and human subjects. Biomechanical studies, cadaveric studies, technique demonstrations and panel discussions were excluded. The ISHA has electronically published and made available the abstracts for all papers presented in the 5 years under study and all posters presented in 2011, 2012 and 2014. Two reviewers independently screened the abstracts of the available presentations. At the end of the reviewing process any disagreements were discussed by the two reviewers until a consensus was reached. The two reviewers then independently evaluated the abstracts and assigned a level of evidence (level I–IV) to each abstract using the AAOS classification scheme [5]. Any disagreements that could not be resolved through discussion between the two reviewers were resolved with input from the senior author.

### Data extraction and statistical analysis

Relevant study data were abstracted from the included presentations, including study type, study location and level of evidence. These data were recorded in Microsoft Excel 2013 (Microsoft, Redmond, WA). In order to assess the inter-reviewer agreement, kappa (κ) was calculated for the abstract screening stage as well as for the presentation evaluation stage. Agreement was categorized *a priori* as follows: κ of 0.61 or greater was considered substantial agreement; κ of 0.21–0.60, moderate agreement; and κ of 0.20 or less, slight agreement. The proportions and frequencies of the levels of evidence were determined for each year. Chi-square tests were used in order to test for non-random trends in the level of evidence presented over the various annual meetings. A *P*-value of 0.05 or less was considered to be significant. All statistics were calculated using Minitab statistical software version 17 (Minitab Inc., State College, PA).

## RESULTS

Of the 582 available presentations presented from 2010 to 2014, 428 were included for assessment. The reviewers in this study reached substantial agreement at the abstract screening and level of evidence evaluation stage with κ (and 95% confidence intervals) of 0.83 (0.79, 0.87) and 0.86 (0.84, 0.88), respectively. Overall, 10.1% of the presentations were level I, 12.8% were level II, 30.1% were level III and 47.0% were level IV evidence for an average level of evidence of III (3.14) over the 5 years evaluated.

The mean sample size of all 428 included presentations was 149 (SD 427) subjects. Although the most frequent type of study design was therapeutic (58.2%), the proportion of therapeutic studies varied depending on the level of evidence of the presentation (Table I). 34.9% of level I, 60.0% of level II, 33.3% of level III and 79.1% of level IV studies were considered therapeutic studies. Prognostic studies were the second most prevalent type of study (31.5%) and diagnostic studies were least common (10.3%). The mean (SEM) sample size by study type for the therapeutic, prognostic and diagnostic presentations was 101 (14), 228 (57) and 154 (56) subjects, respectively.

**Table I.** Total number of presentations by year, level of evidence and type of study for both paper and poster presentations

	**Paper presentations**															***n***
	Level I			Level II			Level III			Level IV			**Totals**			
	T	P	D	T	P	D	T	P	D	T	P	D	T	P	D	
2010	1	2	1	2	0	0	1	5	0	12	4	2	16	11	3	68
2011	3	2	1	2	0	1	2	3	4	14	2	1	21	7	7	95
2012	3	2	0	2	1	1	5	4	2	15	2	0	25	9	3	124
2013	1	3	0	2	1	1	6	6	1	19	4	0	28	14	2	262
2014	3	2	0	5	1	1	8	9	2	11	3	0	27	15	3	131
**Totals**	11	11	2	13	3	4	22	27	9	71	15	3	117	56	15	
***n***	53	135	85	113	135	40	125	188	54	90	427	52	95	246	54	

	**Poster presentations**															
	Level I			Level II			Level III			Level IV			**Totals**			**n**
	T	P	D	T	P	D	T	P	D	T	P	D	T	P	D	

2010	No information available															
2011	1	2	0	5	1	1	3	8	2	25	8	2	34	19	5	134
2012	0	2	0	7	2	2	9	13	9	34	5	1	50	22	12	141
2013	No information available															
2014	3	10	1	8	7	2	9	14	4	28	7	2	48	38	9	183
**Totals**	4	14	1	20	10	5	21	35	15	87	20	5	132	79	26	
***n***	58	180	50	144	202	218	80	140	119	105	389	571	106	211	253	

T, therapeutic study; P, prognostic study; D, diagnostic study and *n*, mean sample size (number of subjects) of the respective presentations.

**Fig. 1.**
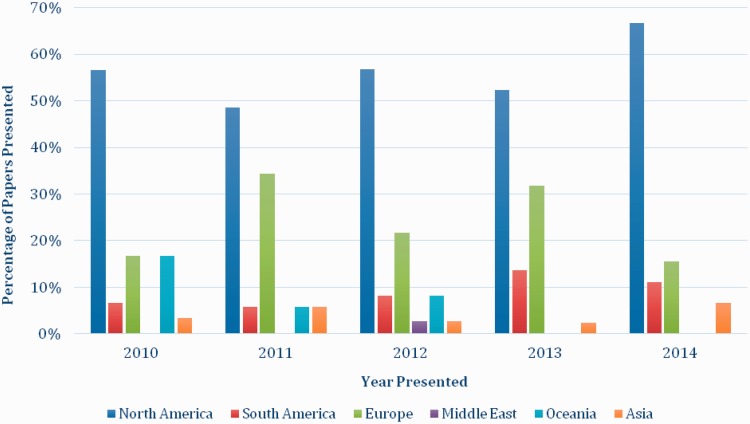
The percentage of paper presentations by geographic location of author and year of presentation. The 2010 ISHA Annual Scientific Meeting took place in Mexico, 2011 in France, 2012 in the United States, 2013 in Germany and 2014 in Brazil.

**Fig. 2.**
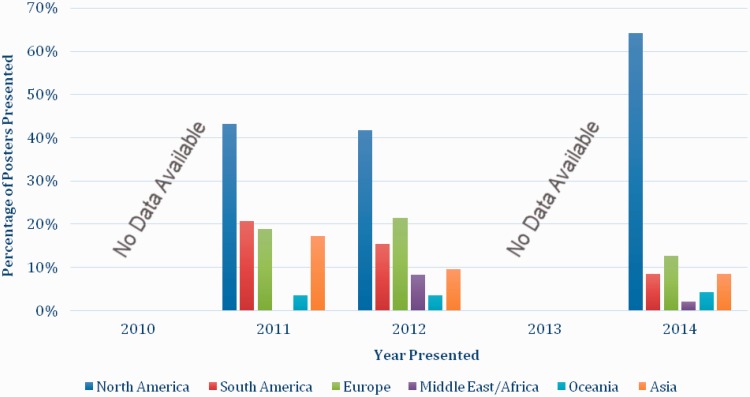
The percentage of poster presentations by geographic location of author and year of presentation. The 2011 ISHA Annual Scientific Meeting took place in France, 2012 in the United States and 2014 in Brazil.

A total of 31 different countries were listed as the country of residence for all presenting authors between 2010 and 2014. There were no significant trends in the number of countries represented at each meeting over the study period. Most of the presenting authors had come from the United States (51.9%), the United Kingdom (9.8%), Chile (5.6%), Australia (4.4%) and Brazil (4.0%) with all other countries contributing <4% of all included presentations (*n* = 428). The regional contribution by year and continent is displayed in Figures 1 and 2 for both poster and paper presentation.

**Fig. 3.**
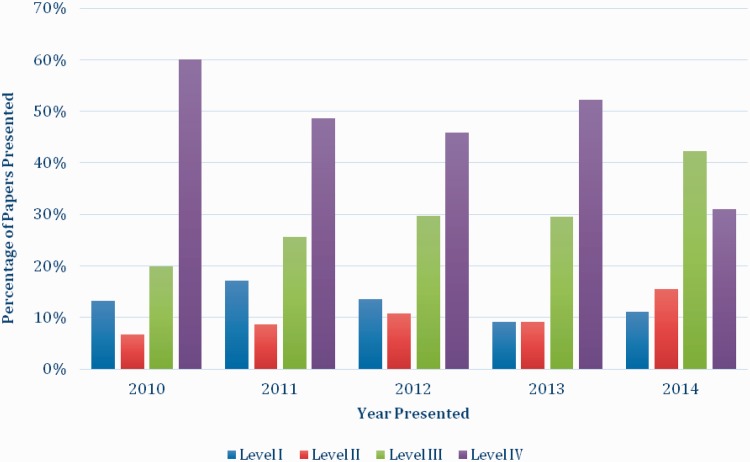
The percentage of paper presentations by level of evidence and year of presentation.

For the paper presentations, the proportion of level IV studies presented decreased by 29% from 2010 (60%) to 2014 (31%) while the proportion of level III studies presented increased by 22% from 2010 (20%) to 2014 (42%) (Fig. 3).

**Fig. 4.**
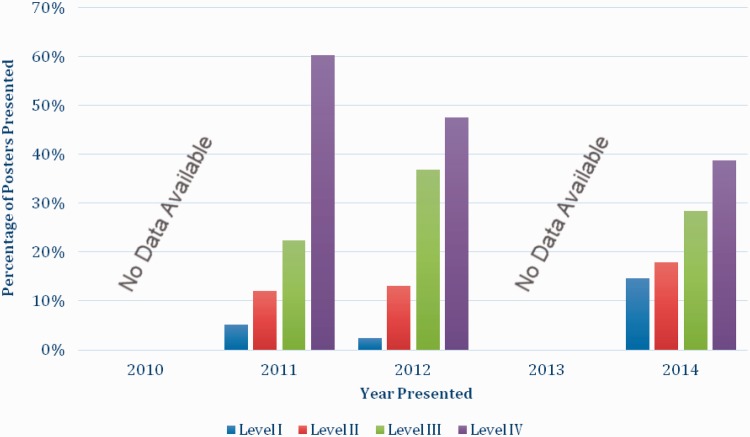
The percentage of poster presentations by level of evidence and year of presentation.

No data was available for the posters presented in 2010 and 2013. The number of level IV studies presented decreased monotonically from 2011 (60%) to 2012 (48%) to 2014 (39%). The number of level II studies increased monotonically from 2011 (8%) to 2012 (13%) to 2014 (18%) (Fig. 4).

For the paper presentations alone, the results of the Chi square analysis indicated that the association between the level of evidence of the presentation and the year presented was not significant (*P* = 0.61). However, the poster presentations (*P* = 0.012), and combined paper and poster presentations (*P* = 0.042) showed a significant, non-random improvement in level of evidence over the meetings 2011, 2012 and 2014 in general. Specific analysis on each level of evidence revealed a significant decrease in level IV studies over this same time period (*P* = 0.036). The number of studies assigned level I, II and III were not sufficient to generate any significant results when evaluated independently.

## DISCUSSION

The ISHA Annual Scientific Meeting is an important venue focused on presenting outcome measures for both common and new procedures in the rapidly changing field of arthroscopic hip surgery. The level of evidence of the research that is presented is significant as physicians are encouraged to take the quality of evidence into account when making their clinical decisions.

In general, the presentations presented at ISHA in the past 5 years predominantly used a study design of lower quality, where 77.1% of all presentations were level III or IV evidence. Level IV studies were the most commonly presented studies, encompassing almost half of all the presentations from 2010 to 2014. Most level IV studies are case series, a descriptive study following a group of patients lacking a control group for comparison. There is a higher likelihood of bias for this study design including selection bias, measurement bias, and the presence of confounding factors. As a result, such studies should not be used to make causal inferences between treatment and outcomes. Although these studies may not be ideal for clinical application, they are nevertheless useful as a method of generating hypotheses of apparent relationships that can be used as the basis for more rigorous study designs [6].

Between 2010 and 2014, the percentage of level IV studies presented decreased, while the percentage of level II and III studies presented increased. This trend in the level of evidence could be explained by the exponential growth in multinational interest in hip arthroscopy and improved understanding of conditions affecting the hip joint [7]. The number of publications relating to femoroacetabular impingement (FAI) increased 5-fold between 2005 and 2010 [8]. As new procedures such as hip arthroscopy for FAI become more common, it is increasingly feasible to design research with higher quality methodology based on hypotheses generated from increasingly available research with lower quality methodology. Thus, the improved quality of the presentations could be attributed to both an increase in the quantity of submissions, allowing for selection of presentations with higher quality methodology, as well as the maturation of the field resulting in a change in the proportion of each type of study produced. The natural progression of a field is to first determine the treatments and procedures that are safe using level IV studies. After this, level II and III studies are undertaken with comparisons to historical or contemporaneous controls. Finally, when a concept is shown to be effective, level I studies, including RCTs, are produced in order to properly validate all findings and associations.

Although the proportion of level II and III presentations increased between 2010 and 2014, there were no such trends indicating an increase in proportion of level I studies. Furthermore, 58.1% of level I studies were prognostic and 7.0% diagnostic indicating that only 3.5% of all studies had a RCT design. The inherent difficulty of performing an RCT study in surgery, particularly for a new procedure, may provide an explanation for the small number of RCTs and why there was no definitive trend indicating an increase in their proportion. In orthopaedic research in general, there are less RCTs than any other study design due to inherent challenges. One important challenge is the inability to blind surgeons to the procedure being performed as well as, in many cases, patient reluctance to randomization. Not only do these challenges affect the quantity of RCTs, but Bhandari et al. has reported that a significant proportion of the RCTs published in *The Journal of Bone and Joint Surgery* lack important tenants of proper methodology such as concealed randomization, reasons for excluding patients and blinding of outcome assessors [9]. Furthermore, hip arthroscopy with its inherent difficulty and prolonged learning curve requires expertise and specialized training that may not be readily available [10]. Although barriers to producing surgical RCTs exist, Farrokhyar and colleagues have reported that RCTs are both vital and viable in novel surgical interventions. Specific attention to proper methodology for such trials, as well as studies assessing their feasibility could aid in producing optimal studies for evidence-based practice [11].

### Strengths and limitations

This study is the first to assess the level of evidence of the paper and poster presentations presented at the ISHA Annual Scientific Meetings. The reviewers in this study had high agreement with respect to the level of evidence evaluations indicating a consistent categorization of the evidence. However, this study is limited by the availability of abstracts (posters missing in years 2010 and 2013) and the fact that abstracts contain limited information (300 word limit). This missing data could have altered the trend in the assessment of quality of study design for the poster presentations.

Overall, the trends generally indicate an improvement in the level of evidence presented at the annual ISHA meeting from 2010 to 2014 with the average level of evidence improving from 3.27 to 2.92. Voleti *et al*. [12] show a significant improvement in the level of evidence of both paper and poster presentations across all orthopaedic subspecialties indicating a more substantial improvement at AAOS annual meetings between 2001 and 2010 with the average level of evidence improving from 3.46 to 2.88. The percentage of higher quality (level I and II) presentations at the ISHA meetings (22.9%) was slightly less than the corresponding percentage at the AAOS meetings (26.7%), but greater than the corresponding percentage of papers presented at The Pediatric Orthopaedic Society of North America annual meeting over the years 2001, 2002, 2007 and 2008 (14%) [12, 13]. All abstracts that are submitted for presentation at the ISHA annual meeting are graded by at least three blinded reviewers with specific grading criteria including sample size and methodology of the study. Although the ISHA Annual Scientific Meeting program committee does an excellent job selecting the highest quality presentations, the results of this process is limited by the quality of research that is submitted. Thus the trends in level of evidence likely indicate an improvement in the quality of research submitted by the authors and continued facilitation of higher quality submissions is vital. Encouraging authors to include levels of evidence with submissions can be helpful; however, Schmidt *et al*. [14] has reported a tendency for authors at AAOS meetings to rate their own presentations with higher levels of evidence than an independent reviewer. Having independent reviewers rate the level of evidence of the research submitted to ISHA scientific meetings could give the audience a sense of the quality of the research being presented and encourage the submission of research with higher methodological quality.

## CONCLUSIONS

The ISHA Annual Scientific Meeting is the premier forum for presenting the latest research from around the world in the novel arena of hip arthroscopy and thus the research presented at these meetings are a surrogate marker for the current research in this field. Although the majority of presentations at the ISHA Annual Scientific Meetings were of lower quality (level III and IV evidence) statistical trends indicate an improvement in the quality of clinical evidence presented from 2010 to 2014. These trends may represent the successful influence of EBM as well as increased multinational interest in hip arthroscopy. Reporting the level of evidence of each presentation and education of authors on proper study methodology could facilitate further improvement in the quality of research presented at these important venues.

## CONFLICT OF INTEREST STATEMENT

Dr M.R.S., one of the authors, has served as program chair for the ISHA Annual Scientific Meetings for the years 2011–2014.
